# Predicting participation of people with impaired vision in epidemiological studies

**DOI:** 10.1186/s12886-018-0889-9

**Published:** 2018-09-04

**Authors:** Pedro Lima Ramos, Rui Santana, Laura Hernandez Moreno, Ana Patricia Marques, Cristina Freitas, Amandio Rocha-Sousa, Antonio Filipe Macedo, The Portuguese visual impairment study group, Amandio Rocha-Sousa, Marta Silva, Sara Perestrelo, João Tavares-Ferreira, Ana Marta Oliveira, Cristina Freitas, Keissy Sousa, Ricardo Leite, José C. Mendes, Andreia B. Soares, Rui C. Freitas, Pedro Reimão, Marco Vieira, Joel Monteiro, Natacha Moreno, Gary Rubin, Ana P. Marques, Rui Santana, Laura Moreno, Pedro L. Ramos, Antonio F. Macedo

**Affiliations:** 1 Low Vision and Visual Rehabilitation Lab, Department and Center of Physics – Optometry and Vision Science, University of Minho, Braga, Portugal; 2 Department of Medicine and Optometry, Linnaeus University, 39182 Kalmar, Sweden; 3 Centro de Investigação em Saúde Pública, Escola Nacional de Saúde Pública, Universidade NOVA de Lisboa, Lisbon, Portugal; 4 Department of Ophthalmology, Hospital de Braga, Braga, Portugal; 5 Department of Surgery and Physiology, Faculty of Medicine, University of Porto, Porto, Portugal; 6 Department of Ophthalmology, Centro Hospitalar São João, Porto, Portugal

**Keywords:** Study participation, Epidemiologic studies, Study design, Vision impairment, Recruitment strategies

## Abstract

**Background:**

The characteristics of the target group and the design of an epidemiologic study, in particular the recruiting methods, can influence participation. People with vision impairment have unique characteristics because those invited are often elderly and totally or partially dependent on help to complete daily activities such as travelling to study sites. Therefore, participation of people with impaired vision in studies is less predictable than predicting participation for the general population.

**Methods:**

Participants were recruited in the context of a study of prevalence and costs of visual impairment in Portugal (PCVIP-study). Participants were recruited from 4 Portuguese public hospitals. Inclusion criteria were: acuity in the better eye from 0.5 decimal (0.30logMAR) or worse and/or visual field of less than 20 degrees. Recruitment involved sending invitation letters and follow-up phone calls. A multiple logistic regression model was used to assess determinants of participation. The J48 classifier, chi-square and Fisher’s exact tests were applied to investigate the possible differences between subjects in our sample.

**Results:**

Individual cases were divided into 3 groups: immediate, late and non-participants. A participation rate of 20% was obtained (15% immediate, 5% late). Factors positively associated with participation included years of education, annual hospital attendance, and intermediate visual acuity. Females and greater distance to the hospital were inversely associated with participation.

**Conclusion:**

In our study, a letter followed by a phone call was efficient to recruit a significant number of participants from a larger group of people with impaired vision. However, the improvement in participation observed after the phone call might not be cost-effective. People with low levels of education and women were more difficult to recruit. These findings need to be considered to avoid studies whose results are biased by gender or socio-economic inequalities of their participants. Young subjects and those at intermediate stages of vision impairment, or equivalent conditions, may need more persuasion than other profiles.

**Electronic supplementary material:**

The online version of this article (10.1186/s12886-018-0889-9) contains supplementary material, which is available to authorized users.

## Background

Epidemiologic studies involve collecting data from large number of individuals. However, participation rates in such studies, particularly in industrialised countries, have been falling in the past 3 or 4 decades. A study in Finland showed a decline in response rates from 84% (men) and 85% (women) in 1978 to 59% (men) and 71% (women) in 2002 [1]. High participation is necessary to ensure, for example, that the participating group is a representative sample of the population. When recruiting fails, statistical power of the results is reduced and conclusions may be distorted [2–5]. In order to produce reliable outcomes, researchers need to consider possible problems arising during the recruitment process and, if possible, control for factors that lead to reduced participation.

During recruitment general and study-specific challenges arise according with the topic and the target population. Some studies have shown that participation rates are influenced by: education (participation increasing with the level of education [6–8]), gender (women tend to participate more than men [9–11]) and marital status (married people participating more than others [12]). Another factor that has been found to influence participation is general health, as given by the index of comorbidities [8]. There are other aspects such as age in which results are less consistent, with some studies showing that older people are more likely to participate [9, 10], whilst others found higher participation rates among young people [6]. Less commonly reported determinants include, for example, ethnicity. In a study by Patel et al. black, asian and other ethnic minorities were less likely to participate [8]. However, in addition to the characteristics of the target group, recruiting strategies can also influence rates of participation.

Previous studies have shown that researchers, when contacting prospective participants, must sound trustworthy and must take into account the motivations of the subject. Slegers and Glass recommend the use of public phone numbers and clear references stating that the study is being carried out by a public institution (when this is the case), in order to increase credibility [13]. They also recommend emphasizing that others invited have already responded to the study call and to provide open, clear and honest information from the onset (e.g. regarding monetary compensation or possible expenses). Personalised letters and reply paid envelopes are also known to improve response rates [14]. Other researchers investigated the primary reasons to take part in epidemiologic studies and concluded that participation is, amongst others, driven by moral reasons [13, 15]. In contrast, the actual effort required to participate has been identified has a barrier. Participation rates are expected to have a negative correlation with the amount of effort that participation requires [16].

The findings mentioned so far have been reported for studies in general; however, there is a lack of information about the profile of people with eye diseases and/or vision impairment (VI) who participate in epidemiological studies. Although, there is one study by Rahi et al. which investigated the engagement of families with children with VI [17]. However, this group was more interested in health service barriers for parents with children with VI [17].

Studies involving directly people with VI have unique characteristics because those invited are often elderly and totally or partially dependent on help to complete daily activities such as travelling to study sites. This makes participation more unpredictable than for many of the studies referred. The purpose of the project from where this study originates was to determine the causes of vision impairment amongst patients attending outpatient eye clinics. In parallel we also wanted to conduct a cross-sectional study about the impact of VI and other clinical and social aspects [18–21].

Our goal with this study was to determine the probability of participation as a function of personal characteristics, including severity of vision loss. We conducted a detailed investigation to distinguish between those who accepted the invitation to take part immediately from those who needed further contact before agreeing to participate. According to the “continuum of resistance” model, the more contacts a subject requires in order to take part in a study, the more similarities he/she shares with non-participants [16, 22]. The participation model was tested in our sample by comparing those that agreed to participate with non-participants.

We hypothesized that: i) the lower the acuity is the less likely participation is; ii) participation is independent of the cause of VI; iii) participation is affected by the distance residence-hospital; iv) education increases participation; v) age and gender affect participation; vi) annual hospital attendance increases participation.

To our knowledge this is the first study to investigate participation rates and its determinants in research involving people with VI. By studying participant’s profiles, we hope to provide a significant contribution to the scientific community when planning studies involving people with VI and similar conditions.

## Methods

### Study design

The prevalence and costs of visual impairment in Portugal (PCVIP-study) was a hospital-based study whose aim was to determine, prevalence, causes and costs of VI in Portugal. The study gathered demographic, clinical, and economic information of people with VI. Participants for this report were recruited at 4 Portuguese public hospitals; patients with VI attending outpatient appointments at each of the hospitals for a period of 12 months were invited to participate in the study. The inclusion criteria were: patients with visual acuity (VA) in the better eye of 0.5 decimal (0.30logMAR) or worse and/or visual field less than 20 degrees. Cases were entered in a database by qualified and trained clinical staff. The database is online at http://www.pcdvp.org/login.php. The study protocol required inviting patients to attend an in-hospital appointment with the research team for face-to-face interviews and additional visual measurements. The study was designed considering the recommendations of the Vancouver Economic Burden of Vision Loss Group [23]. Basic demographic information was collected from administrative databases at the hospital. Information included: subject’s initials, date-of-birth, gender, and place of residence (*“concelho”*, in Portuguese, equivalent to district in many countries).

### Participants

Letters were posted using the hospital mail service, the logo of the hospital was printed on the envelope and letters were sent directly to the patients’ address. All documents were printed in font Arial- 16 point. The mail envelope included a letter of invitation signed by a physician from the local hospital (1 page), an information booklet (3 pages), a consent form (1 page) and a reply-paid envelope addressed to Escola Nacional de Saúde Publica, Lisboa (National School of Public Health, Lisbon). Information was printed on both sides of the paper; consent forms were printed on the reverse side of the invitation letter. In addition to information about investigators, institutions, contact details and clinicians involved in the study the letter contained a clear and isolated sentence (in Portuguese) with the instruction: *“If you agree to take part in this study, please tick the boxes in the flipside of this sheet, sign at the bottom of the page and provide a valid contact number for us to book your appointment at the hospital”.*

If a response was not received within 2 weeks, a follow-up phone call was made. Calls were made by an experienced hospital staff member trained and informed about the study with instructions to ask the following questions: i) *did you receive our letter?* ii) *If yes, can I provide any further information about the study and the letter?* iii) *Would you be interested in taking part in this study?* If the person declined the invitation to participate, they were asked questions about: 1) years of education; 2) marital status; 3) annual hospital attendance.

For positive respondents, an appointment was booked at the hospital where they normally receive eye care and the same information was obtained. Those that agreed to take part in the study are defined as “participants” and those that declined after all attempts are defined as “non-participants”. Those that dropped out after initially agreeing were not included in either of these categories. Participants were divided into 2 sub-grougps: “immediate participants” - those who sent the reply paid envelope with the consent form without being contacted by phone, and “late participants” - those who agreed to take part in the study only after they were contacted by telephone.

### Data analysis

A database was built with information about: age, gender, distance between residence and hospital where the participant was recruited (DISTH), years of education (EDU), marital status (MST), visual acuity in the better eye (VA), annual hospital attendance (AHATTEND), cause of vision impairment (CAUSE-VI), Charlson comorbidities index (CCI). Information about causes of vision impairment and comorbidities to compute CCI was retrieved from medical records. The CCI measures to which extent an individual is affected by comorbidities [24].

Univariate differences in participation rates according to the independent categorical variables were assessed using chi-square tests. DISTH, EDU and CCI are, unless otherwise stated, continuous variables and the remainder are categorical. Multiple logistic regression (R data analysis software, v3.2.4 for Windows) was used to determine the effect of independent variables in participation rates. The final model was built upon a database with 600 individuals and the fit quality was firstly measured also within such database. That is, the sample was both the training data and the testing data. In addition, an internal validation of this model was performed, a 10-fold cross-validation using the logistic classifier of Weka 3.8.

## Results

For the current study a group of 2130 individuals were contacted by letter. Of the initial 2130 letters sent, 31 were returned to sender and 349 individuals agreed to participate immediately (17% of 2099). Of these, 49 individuals eventually dropped out of the study for health reasons or transportation difficulties (the study only covered travel expenses up to 15 euro), this resulted in 300 immediate participants (15% of 2050).

Phone calls were made to 1750 non-respondents in order to invite them to participate; 89 were unreachable by phone. From the 1661 contacted by telephone 84 (5%) agreed to take part. Therefore, the final number of participants was 384 (20%) out of 1961 that could be successful reached by letter and/or phone call.

In total, 600 individuals (260 females or 43%) with a mean age of 66 years (SD = 16.7) were included in this sample. In our analysis 325 (54%) were participants and 275 (46%) were non-participants. Non-participants analysed are a random sample of the total (1577) selected from successive cases in our list with all the required information. From the 384 participants only 325 were included in this report because the remaining 59 were waiting for the interview.

The median CCI for the entire sample was 0.6 (IQR = 1.8), amongst participants was 0.8 (IQR = 1.75), for non-participants was 0.5 (IQR = 1.5); this difference was not statistically significant (Mann-Whitney, U = 1110, *p* = 0.45).

The median EDU in years for the complete sample was 4 (IQR = 3), for participants was 4 (IQR = 5), for non-participants was 4 years (IQR = 1); this difference was statistically significant (Mann-Whitney, U = 63,752, *p*-value < 0.001). The number of years of education can be considered low but is expected for the age and geographical location of the participants [25].

The median DISTH (in kilometres) for the complete sample was 9.6 km (IQR = 24.2), for participants was 1 km (IQR = 15.1) and for non-participants was 19.4 km (IQR = 38.7); this difference was statistically significant (Mann-Whitney, U = 24,416, *p* < 0.001). Other socio-demographic and VI-related data are summarized in Tables 1 and 2.

**Table 1:** Summary of the distribution of 600 subjects included in the analysis. Among 600, 325 are participants ^a^ (immediate or late) and 275 non-participants randomly selected from 1577 total non-participants

Characteristic	*n* (%)	Participation YES/NO	Participation (%)	*p*-value (χ^2^)
Gender				< 0.001
Male	339 (56.6)	225/114	66.4	
Female	261 (43.4)	100/161	38.3	
Age group				0.00535
< 20 yrs	14 (2.3)	12/2	85.7	
20 to < 30 yrs	8 (1.3)	6/2	75.0	
30 to < 40 yrs	28 (4.7)	27/1	96.4	
40 to < 50 yrs	43 (7.2)	34/9	79.1	
50 to < 60 yrs	82 (13.7)	52/30	63.4	
60 to < 70 yrs	137 (22.8)	80/57	58.4	
≥ 70 yrs	288 (48.0)	114/174	39.6	
Number of Hospital Appointments per year (AHATTEND)				< 0.001
Low - AHA (≤4×/yr)	173 (28.8)	52/121	30.1	
Medium - AHA (5 to 9×/yr)	178 (29.7)	86/92	48.3	
High – AHA (≥ 10×/yr)	249 (41.5)	187/62	75.1	
Marital Status (MST)				< 0.001
Married	261 (43.5)	110/151	42.1	
Living together	85 (14.2)	76/9	89.4	
Single	82 (13.7)	56/26	68.3	
Widow	131 (21.8)	48/83	36.6	
Divorced	41 (6.8)	35/6	85.4	
Visual Acuity- decimal scale (VA)				< 0.001
0	42 (7.0)	26/16	61.9	
0.1	80 (13.3)	51/29	63.8	
0.2	105 (17.5)	43/62	40.9	
0.3	87 (14.5)	35/52	40.2	
0.4	129 (21.5)	63/66	48.8	
0.5	157 (26.2)	107/50	68.1	
Aetiology of visual impairment (CAUSE-VI)				0.4336
Adult Macular Degeneration	76 (16.0)	31/45	40.8	
Diabetic retinopathy	191 (40.1)	110/81	57.6	
Glaucoma	60 (12.6)	26/34	43.3	
Other	149 (31.3)	81/68	54.4	
Multiple or undefined	124			

^a^Participants as mentioned here include immediate and late participants

**Table 2:** Summary of the distribution of all cases (*n* = 600) according to participation

Characteristic	Participants (*n* = 325)		Non-participants (*n* = 275) *n* (%)	*p*-value (χ^2^)
	Immediate (*n* = 241) *n* (%)	Late (*n* = 84) *n* (%)		
Gender				< 0.001
Male	183 (75.9)	42 (50)	114 (41.4)	
Female	58 (24.1)	42 (50)	161 (58.6)	
Age group				0.00535
< 20 yrs	10 (4.1)	2 (2.4)	2 (0.7)	
20 to < 30 yrs	2 (0.8)	4 (4.8)	2 (0.7)	
30 to < 40 yrs	14 (5.8)	13 (15.5)	1 (0.4)	
40 to < 50 yrs	27 (11.2)	7 (8.3)	9 (3.3)	
50 to < 60 yrs	43 (17.8)	9 (10.7)	30 (10.9)	
60 to < 70 yrs	64 (26.6)	16 (19)	57 (20.7)	
≥ 70 yrs	81 (33.7)	33 (39.3)	174 (63.3)	
Number of Hospital Appointments per year				< 0.001
Low - AHA (≤4×/yr)	42 (17.4)	10 (11.9)	121 (44)	
Medium - AHA (5 to 9×/yr)	70 (29)	16 (19)	92 (33.5)	
High – AHA (≥ 10×/yr)	129 (53.6)	58 (69)	62 (22.5)	
Marital Status				< 0.001
Married	75 (31.1)	35 (41.7)	151 (54.9)	
Living together	76 (31.5)	0 (0)	9 (3.3)	
Single	35 (14.5)	21 (25)	26 (9.5)	
Widow	25 (10.4)	23 (27.3)	83 (30.2)	
Divorced	30 (12.4)	5 (6)	6 (2.1)	
Visual Acuity (decimal scale)				< 0.001
0	18 (7.5)	8 (9.5)	16 (5.8)	
0.1	28 (11.6)	23 (27.4)	29 (10.5)	
0.2	33 (13.7)	10 (11.9)	62 (22.5)	
0.3	31 (12.9)	4 (4.8)	52 (18.9)	
0.4	47 (19.5)	16 (19)	66 (24)	
0.5	84 (34.8)	23 (27.4)	50 (18.3)	
Aetiology of visual impairment(*)				0.4336
Age-related Macular Degeneration	18 (7.5)	13 (15.5)	45 (16.4)	
Diabetic retinopathy	87 (36.1)	23 (27.4)	81 (29.5)	
Glaucoma	17 (7.1)	9 (10.7)	34 (12.4)	
Other	58 (24.1)	23 (27.4)	68 (24.7)	
Multiple or undefined	124			

### Factors predicting participation using a logistic regression model

All the results reported in this section compare participants (the group who agreed to take part in the study after an invitation letter or letter and a follow-up phone call) with the cases of non-participants (the group of cases that declined after both contacts). We used a diagnostic test for the multicollinearity of predictors, the variance inflation factor, calculated for each predictor. The highest inflation factor was 1.67 for AHATTEND. Which means that AHATTEND was slightly correlated with the other predictors; nevertheless, this value was below the critical value of 2.5 reported in the literature as the tolerable upper limit [26].

In an initial model, with a binary dependent variable that assigned a value of 1 to “participants” and 0 to “non-participants”, some variables were independent predictors of participation (see Additional file 1: Table S1).

Amongst categorical predictors we found an effect for gender (males participated more, *p* < 0.001), AHATTEND (participation for AHA-high was different from participation for AHA-low, *p* < 0.001), MST (co-habiting, single or divorced individuals were more likely to participate than married individuals, *p* < 0.001), VA (individuals with VA of 0.2 or 0.3 were less likely to participate than blind individuals, *p* < 0.001) and CAUSE- VI (individuals with diabetic retinopathy were more likely to participate than individuals with AMD, *p* = 0.03).

Amongst continuous predictors we found statistically significant effects for DISTH (participation reduced with increasing distance, *p* < 0.001) and EDU (participation increased with the number of years of education, *p* < 0.001).

The initial set of levels for each categorical variable were based on authors’ experience (see Additional file 1: Table S1). For the final model, non-significant variables were removed and other levels or categories were defined as summarized in Additional file 2: Table S2. We now give an example to explain the rational. In the initial model, Additional file 1: Table S1, we observed that the effect of “Medium-AHA” in participation was not statistically different (*p* = 0.075) from the reference category “Low-AHA”, therefore we merged these 2 categories and re-classified cases as “AHA-rare”, Additional file 2: Table S2. Cases classified as “High-AHA” in the first model were kept separately because there was a statistically significant effect of this category in the model (*p* < 0.001). This category was renamed “AHA-frequent” to be consistent with the other category of the variable AHATTEND.

The variance inflation factor was recalculated for each predictor. The highest value obtained was 1.079 for MST, which means that multicollinearity can be ignored. Results for the final model are summarized in Table 3. All independent variables considered had a significant effect on the dependent variable. The deviance chi-squared goodness of fit test confirmed an excellent fit of the model to the data (*p*-value = 0.99).

**Table 3:** Multivariable logistic regression model used to predict the probability of participation

Variables/Characteristic	Beta coefficient (SE)	Odds Ratio (95% CI)	*p*-value
Gender			< 0.001
Female vs. Male	−1.27 (0.24)	0.28 (2.23–5.71)	
Distance to clinic - km (DISTH)	−0.02 (0.004)	0.98 (1.01–1.03)	< 0.001
Education – years (EDU)	0.21 (0.04)	1.23 (1.14–1.33)	< 0.001
Annual number of hospital visits - in times-per-year (AHATTEND)			< 0.001
≥ 10×/yr vs < 10×/yr	1.64 (0.24)	5.18 (3.24–8.69)	
Marital Status (MST)			< 0.001
Living together vs. Others (married, single or widowed)	3.26 (0.46)	26.14 (10.62–64.4)	
Divorced vs. Others (married, single or widowed)	2.74 (0.56)	15.44 (5.15–46.27)	
Visual acuity (VA)			< 0.001
Intermediate (0.2–0.4) vs. extreme (0, 0.1 or 0.5)	1.10 (0.23)	3.02 (1.92–4.74)	

*SE* standard error, *CI* Confidence Interval

The likelihood of participation increased if individuals were male, had AHA-frequent, had VA-extreme, if they were co-habiting or were divorced, with more EDU and less DISTH. Formula 1 and Formula 2 summarize these results:

1 $$ Linear\ predictor=-1.71-1.27\ \left( If\ Gender={}^{"}{female}^{"}\right)-0.02 DISTH+0.21 EDU+1.64\left( If\ AHATTEND={}^{"}{frequent}^{"}\right)+3.26\left( If\  MST={}^{"} co-{habiting}^{"}\right)+2.74\left( If\  MST={}^{"}{\mathrm{divorced}}^{"}\right)+1.1\ \left( If\  VA={}^{"}{\mathrm{extreme}}^{"}\right) $$

2 $$ Participation\ probability=\frac{e^{linear\ predicto r}}{1+{e}^{linear\ predicto\mathrm{r}}} $$

A 10-fold (10 iterations) cross-validation of the prediction model was performed. Before the iteration the Weka 3.8 software splits the 600 cases into 10 subsamples (60 cases each). For each iteration, during the validation process, each sample was chosen, at random, once as “testing data”. The remainder 9 (540 cases) were used to generate temporary models. The 10 temporary models were then averaged to generated the final theoretical model which was tested against the real participation results for the 600 cases. The coefficients of the resulting theoretical model were very similar to those summarized in Table 3. The theoretical model classified correctly 484 out of 600 cases, with a weighted average precision of 0.809, a weighted average F-Measure of 0.808 and a weighted average ROC area of 0.872. If taken together the results of the internal validation and the deviance chi-squared goodness of fit, we can say that the model fits the real data accurately.

**Table 4:** Categories used to analyse differences between immediate (Ipar) and late participants (Lpar) and between late and non-participants (Npar)

AGE	AGE_1_ = age less than 40 years AGE_2_ = age between 40 and 69 years AGE_3_ = age 70 or more years
AHATTEND	AHA-rare = number of annual hospital appointments less than 10 AHA-frequent = number of annual hospital appointments 10 or more
EDU	EDU_1_ = less than 12 years of education EDU_2_ = 12 or more years of education
DISTH	DISTH_1_ = if distance residence-hospital was less than 40 Km DISTH_2_ = if distance residence-hospital was 40 Km or more
VA	VA-extreme; includes VA of 0.0 or 0.1 or 0.5 VA-intermediate; includes VA of 0.2 or 0.3 or 0.4
MST	1 = Married; 2 = Together; 3 = Single; 4 = Widow; 5 = Divorced
GENDER	1 = Male; 2 = Female

Table 3 provides the odds ratios (ORs) for study participation. It can be observed that, for example, the odds of a man participating in the study was 3.57 times higher than the odds of a woman.

The model expressed in Formula 1 and Formula 2 was simulated using Matlab (v2014b, Matworks inc.). The simulation allows the visualization of the probability of participation estimated by the model for extreme cases.

**Fig. 1:**
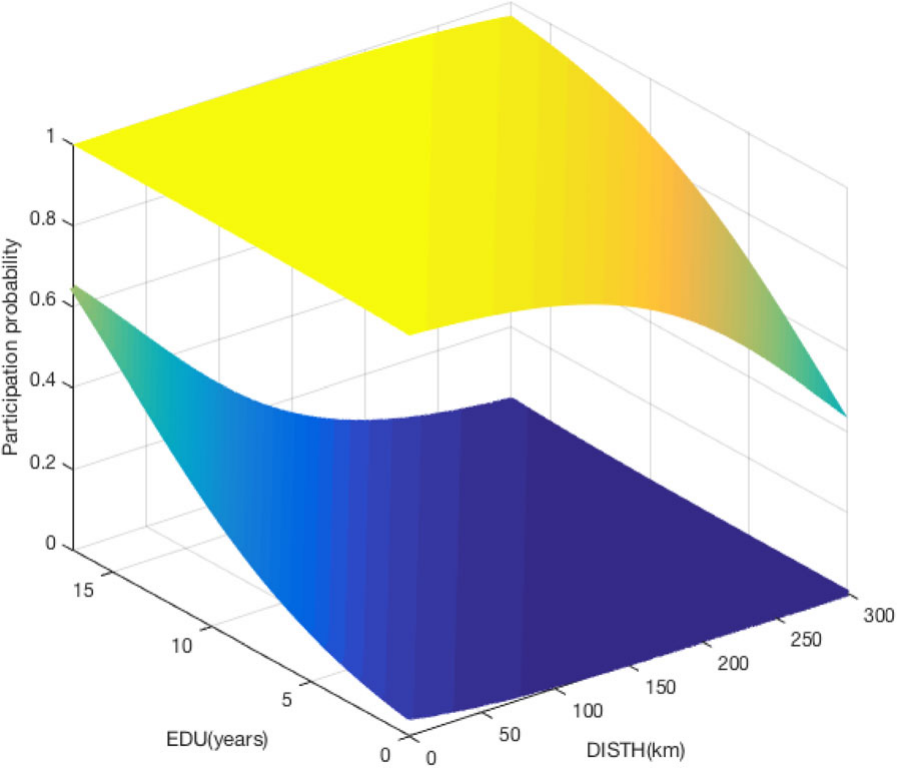
Variation of the probability of participation predicted by our model according with the continuous variables DISTH and EDU. The two surfaces represent the most favourable and less favourable participation profiles defined according with the categorical variables used. The top yellow surface represents a male, with AHA-frequent, living together, with VA-extreme. The bottom blue surface represents a female, with AHA-rare, married, single or widow, with VA-intermediate

According with the final model, the worst profile regarding the probability of participation, was being female attending the hospital 10× or less a year, married, single or widowed, with VA 0.2–0.4. The best profile was being male; attending the hospital 10× or more a year, living in a non-marital partnership, and VA ≤0.1 or 0.5. The model was implemented for these two situations as a function of the continuous variables distance residence-hospital (DISTH) and education in years (EDU), the results are shown in Fig. 1.

In both cases the probability of participation increases when the distance residence-hospital decreases and education increases.

For the best profile and for distances 0-150 km, the participation probability reduces slowly. That is, the distance residence-hospital is almost irrelevant within the range 0-150 km. For distance values greater than 150 km the probability of participation decreases sharply. When living over 150 km away from the hospital, distance would be a big barrier for participation, in particular for those with less than 10 years of education.

Amongst individuals with the worst profile for participation, the distance residence-hospital had little impact for those with less than 10 years of EDU; for EDU greater than 10 years the distance residence-hospital is an important factor for participation when is below 100 km.

The group with the best profile would always have a minimum participation probability of approximately 40% and the worst profile group a maximum participation probability of approximately 60%.

### Comparison between immediate participants (Ipar) and late participants (Lpar)

Here we report results of a comparison between two sub-groups of participants (participants = Ipar+Lpar). Ipar = accepted to participate when invited by letter only; Lpar = accepted to participate after letter followed by a phone call.

We found that the percentage of Lpar+Ipar was significantly higher than Ipar only (McNemar’s test, *p* < 0.001). This shows that the number of participants increased significantly after the follow-up phone call. We investigated if there was a difference between Ipar and Lpar for the demographic aspects summarized in Table 4.

**Fig. 2:**
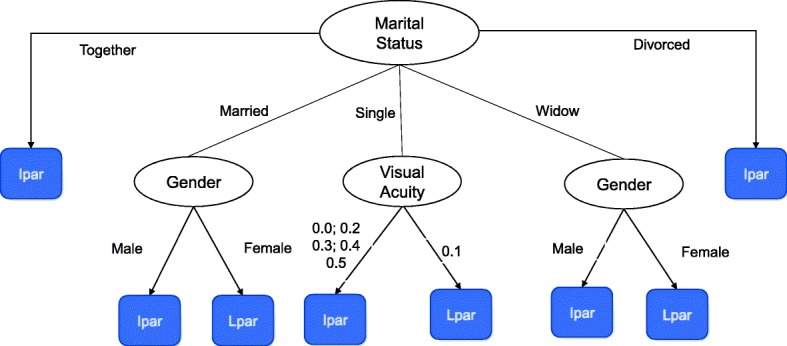
Classification tree originated by the C4.5 / J48 algorithm predicting immediate and late participation

To build the categories defined in Table 4, first we investigated the existence of optimal cut points for the variables using the J48 classifier (Weka 3.8). The resultant decision tree is shown in Fig. 2 - in which the oval nodes represent random variables and rectangular nodes represent decisions or predictions. This classification model has a weighted average precision of 0.821, a weighted average F-Measure of 0.813 and a weighted average ROC area of 0.792. With this method we can predict, for example, that a widow man will be an immediate (Ipar) instead of a late (Lpar) participant. It also predicts that an individual that is single and has VA of 0.1 will be a Lpar instead of an Ipar.

The decision about which demographic aspects would be compared was based on 3 criteria applied according with the sequence presented here: (1) specific hypothesis that the researchers wanted to test, (2) the cut-off points resulting from the J48 classifier analysis and (3) the number of subjects in each category.

The percentage of males in the Ipar was 76% (183 of 241) and in the Lpar was 50% (42 of 84); the distribution by gender was different in both groups (chi-square = 20.21, df = 1, *p* < 0.001).

The percentage of males in the AGE_1_ group was 12% (22 of 183) amongst Ipar and 40% (17 of 42) amongst Lpar (chi-square = 19.3, df = 1, *p* < 0.001, after Bonferroni adjustment). For males with AGE_2_, the percentage was 56% (102 of 183) amongst Ipar and 31% (13 of 42) amongst Lpar (chi-square = 7.3, df = 1, *p* = 0.006, after Bonferroni adjustment).

The percentage of participants with AHA-rare within the group of those who are males and AGE_2_ was 46% (47 of 102) amongst Ipar and 15% (2 of 13) amongst Lpar (Fisher’s exact test, *p* = 0.04).

The percentage of participants with EDU_1_ within the group of those who are females, AGE_2_ and AHA-frequent was 95% (18 of 19) amongst Ipar and 60% (6 of 10) amongst Lpar (Fisher’s exact test, *p* = 0.036).

### Comparison between late participants (Lpar) and non-participants (Npar)

**Fig. 3:**
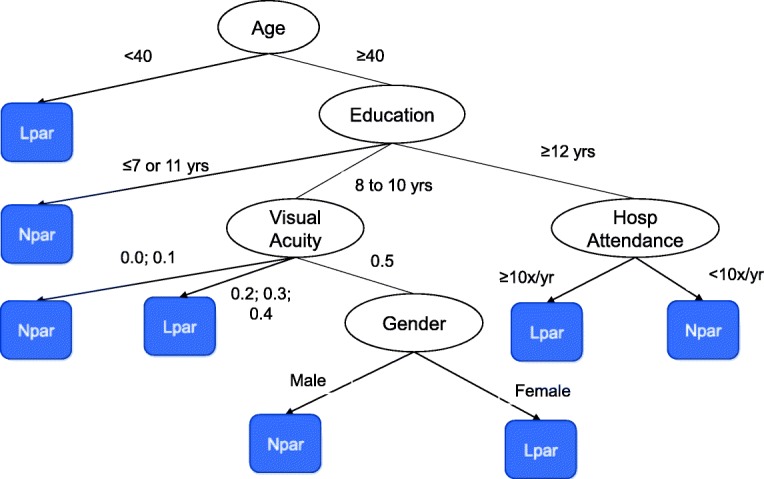
Classification tree originated by the C4.5 / J48 algorithm predicting late participation and non-participation

Here we report an analysis comparing Lpar with Npar (Npar = those decline participation after two invitations). We wanted to investigate if the the profile of Npar and Lpar was similar. If that was true the percentage of cases in each demographic category should be similar in both sub-groups. This analysis is similar to the one performed in the previous section. The J48 classifier originated the decision tree shown in Fig. 3.

This classification model has a weighted average precision of 0.801, a weighted average F-Measure of 0.803 and a weighted average ROC area of 0.688. The classifier predicts that someone younger than 40 years that is not an Ipar will be a late participant (LPar) instead of a non-participant (NPar).The classification tree was used to define the levels summarized in Table 4. It was upon these levels that differences between Lpar and Npar were formally investigated.

The first finding was a difference in age between Lpar and Npar. The percentage of individuals with AGE_1_ was 20% (17 of 84) amongst Lpar and was 2% (5 of 275) amongst Npar (Fisher’s exact test, *p* < 0.001). For those in the group AGE_3_ the proportion was 39% (33 of 84) amongst Lpar and 63% (174 of 275) amongst Npar (chi-square = 12.82, df = 1, *p* < 0.001). The percentage of DISTH_1_ subjects within the group of those who are AGE_2_ was 97% (32 of 33) in Lpar and 78% (76 of 98) in Npar (Fisher’s exact test, *p* = 0.009).

The percentage of individuals with EDU1 within the group AGE_2_ was 73% (24 of 33) in Lpar and 98% (96 of 98) in Npar (Fisher’s exact test, *p* < 0.001). The percentage of individuals with EDU_1_ within the group of those who are AGE_3_ was 88% (29 of 33) in Lpar and 96% (167 of 174) amongst Npar (Fisher’s exact test, *p* = 0.013).

The percentage of AHA-rare subjects within the group of those who are AGE_2_ was 9% (3 of 33) in the Lpar group and 45% (44 of 98) in the Npar group (Fisher’s exact test, *p* < 0.001).

The percentage of individuals AGE_3_ and AHA-rare was 18% (6 of 34) in the Lpar group and 45% (77 of 172) in the Npar group. (Fisher’s exact test, *p* = 0.004).

The percentage of VA-extreme subjects within the group of those who are AGE_1_ was 76% (13 of 17) in Lpar and 20% (1 of 5) in Npar (Fisher’s exact test, *p* = 0.039). The percentage of VA-extreme subjects within the group of those who are AGE_2_ was 61% (20 of 33) in Lpar and 35% (34 of 98) in Npar (chi-square = 6.84, df = 1, *p* = 0.009). The percentage of VA-extreme subjects within the group of those who are AGE_3_ was 64% (21 of 33) in Lpar and 33% (58 of 174) in Npar (chi-square = 9.44, df = 1, *p* = 0.002).

Non-participants were asked to specify reasons for non-participation and the most commonly mentioned reasons were:

- *“I am too debilitated to participate”*
- *“It is far away from my home”*
- *“There are no benefits in participating”*
- *“I have no one to go with me”*

## Discussion

In this study we investigated participation rates in the PCVIP study and its determinants. We obtained an overall participation rate of 20%, low participation was anticipated given that the target group of the population were people with impaired vision. Some that were willing to take part in interviews were not able to participate because travel arrangements were too expensive compared with the compensation offered by our study. Despite this, the participation rate was comparable to other studies involving participation in phone interviews in the Portuguese population [27]. Correia et al. were only able to interview 21.7% of those eligible for their study. When we analysed factors or determinants that are likely to affect participation rates in our study, we found that people at the extremes of VA (≤0.1 or less and 0.5) were more likely to participate than those with intermediate acuities (0.2–0.4). Participation was independent of age and cause of VI but influenced by gender (males were more likely to participate). People living together or divorced were more likely to participate than those in other categories of marital status. Participation reduced with increasing traveling distances to the hospital but increased with the number of years of education. A high frequency of hospital appointments was also favourable to participation. A decision to participate was independent of the Charlson comorbidities index.

The initial hypothesis regarding the effect of acuity was partially confirmed and we were also able to confirm that the cause of VI was not a determinant of participation. Other results are in line with our initial hypotheses, specifically, we confirmed an effect of education, distance to the hospital and frequency of hospital attendance as determinants of participation in our study. Our model predicts that, for individuals with the best profile favouring participation, a minimum of 4 in 10 contacted would participate. For the worst profile, the maximum participation would be 6 out of 10. These profiles need to be considered when designing studies and planning recruitment.

Surprisingly subjects with severe vision loss, acuity 0.1 or less, were more likely to participate than those with better acuity, VA in the range 0.2–0.4. This finding seems to contradict the idea that the sustained willingness of individuals to participate can be inferred from the effort that participation requires [16]. It would be expected, from the effort perspective, that someone with a worse acuity would have more difficulties participating than someone with better acuity. A possible explanation is that individuals at more advanced stages of their conditions may perceive a greater benefit in responding to study participation than those at less advanced stages. People at more advanced may have a stronger moral drive to help others in a similar situation [28]. Another explanation for this result can be the level of adjustment to vision loss. Individuals with worse acuity might be better adjusted to vision loss whilst those in the medium range may still be in the process of adjusting and; therefore, less inclined to participate [29, 30].

The participation rate in our study was higher amongst men than women, which contrasts with some studies [9–11]. This is a result that needs further investigation but we acknowledge that this might be related to cultural factors because Correia et al. also found, in Portugal, higher participation amongst men [27]. Another result that is in contrast to other studies was the higher participation amongst subjects that were divorced or single when compared with married individuals. In a study by Sahar and colleagues married people were more likely to participate than people with other marital status [12]. We do not have a clear explanation for this result, but it could be related to the spectrum of relationships of the target group of the population.

Factors such as distance to the hospital, education or annual hospital attendance are important when planning recruitment. Individuals living further away from the hospital were less likely to participate. This result seems to be explained by the “principle of the effort” that predicts an inverse relationship between effort and participation probability [16]. In line with our results for education status, increased participation with the number of years of education has been reported in other studies [6–8]. The most likely reason for this is the ability to understand the purpose of the study and the contribution that studies can provide to the progress of knowledge. The participation odds for people visiting the hospital 10 or more times per year were higher than the participation odds of those who attend the hospital less than 10 times per year. Differences are likely to be due to the development of an acute civic awareness and/or familiarity with the hospital environment amongst those visiting the hospital more frequently.

In this study we also looked at systematic differences between immediate and late participants. This analysis provides information regarding the spectrum of individuals in which a follow-up phone call can be effective. Overall, we can say that the phone call, as others have found, seems to be important in increasing the moral obligation to participate [13, 15]. Our operators noted that a substantial number of individuals changed their minds and eventually decided to take part in the study after the importance of their participation has been emphasized. Compared with the initial letter, the follow-up call captured more women, more males younger than 40 years but fewer males within the age 40–69 years. Groups in which participation increased need more incentives or clarification than the groups that did not change in participation. Our results are in agreement with other studies showing that Lpar tend to be younger than Ipar [31, 32]. Other differences between Ipar and Lpar that we found involve very small groups with specific characteristics that seem to show only scattered combinations of patterns of participation.

By comparing late participants (Lpar) with non-participants (Npar), we investigated if the model of “continuum of resistance” was valid in our sample. According with the “continuum of resistance” model the more contacts an individual requires to participate in a study the more similar he/she is to Npar [16, 22]. However, similar to results in other studies [33, 34], we found many differences between the structure of the group of Lpar and Npar. In particular, the age distribution was different, Lpar were younger than Npar [31, 32]. Overall, there were several differences between the structure of the group Lpar and Npar which somewhat contradicts what would be expected from the model “continuum of resistance” [31–33, 35].

A limitation of our study was the lack of information concerning the economic status of the subjects that could potentially clarify some of the unexplained findings. Another aspect that we believe would strengthen our results would be the inclusion of responses from more subjects in both groups. Amongst others reasons, some non-participants were excluded from the analysis because they were unable to answer our questions by telephone (for example due to dementia, staying in nursing homes, hospitalization) or the clinical information was of poor quality (to determine, for example, the Charlson comorbidities index). Therefore, the included cases may be slightly different from the general population of interest.

## Conclusion

In conclusion, participation rates in our study were influenced by gender, distance to the hospital, number of years of education, annual hospital attendance, marital status and visual acuity. There were considerable differences between immediate participants and late participants and between late participants and non-participants. Individuals with low levels of education and women were more difficult to recruit. These facts need to be taken in consideration in order to avoid studies that are biased by gender or socio-economic inequalities of the participants. Young subjects and those at intermediate stages of vision impairment, or equivalent conditions, might need more persuasion than other profiles.

## Additional files

Additional file 1: **Table S1.** Initial regression model used. (DOCX 20 kb)

Additional file 2: **Table S2.** The table summarizes new categories that were defined after having run the first logistic regression. The categories were used for our final model. (DOCX 17 kb)

Additional file 3: Data used and analysed in this study. (XLSX 101 kb)

## Abbreviations

AGE_1_: Age less than 40 years
AGE_2_: age between 40 and 69 years
AGE_3_: age 70 or more years
AHA-frequent: number of annual hospital appointments 10 or more
AHA-rare: number of annual hospital appointments less than 10
AHATTEND: annual hospital attendance
AMD: age-related macular degeneration
CAUSE-VI: cause of visual impairment
CCI: Charlson comorbidities index
DISTH: distance from the residence to the hospital
DISTH_1_: distance residence-hospital was less than 40 km
DISTH_2_: distance residence-hospital was 40 km or more
VA: extreme includes VA of 0.0 or 0.1 or 0.5
VA: intermediate includes VA of 0.2 or 0.3 or 0.4
EDU: number of years of education
EDU_1_: less than 12 years of education
EDU_2_: 12 or more years of education
Ipar: immediate participants
Lpar: late participants
MST: marital status
Npar: non-participants
PCVIP-study: Prevalence and Costs of Visual Impairment in Portugal: a hospital based study
SD: standard deviation
SE: standard error
VA: visual acuity in the better eye
VI: vision impairment
