# Supplementary material for: Predicting participation of people with impaired vision in epidemiological studies
Source: BMC Ophthalmol. 2018 Sep 4;18:236. doi: 10.1186/s12886-018-0889-9 (PMC6123934; doi:10.1186/s12886-018-0889-9)
Supplement: Supplementary file 1 — Table S1. Initial regression model used. (DOCX 20 kb) [file 12886_2018_889_MOESM1_ESM.docx]

**Table S1 - Initial regression model used.**

| **Variables** | **Beta estimate** | **Std.error** | **p-value** |
| --- | --- | --- | --- |
| **Intercept** | -1.27 | 1.44 | 0.377 |
| ***Gender: Male** |  |  |  |
| Female | -1.04 | 0.29 | <0.001 |
| ***Age: <20** |  |  |  |
| [20,30[ | -0.76 | 1.73 | 0.66 |
| [30,40[ | 2.12 | 1.62 | 0.189 |
| [40,50[ | 0.36 | 1.33 | 0.328 |
| [50,60[ | 0.67 | 1.29 | 0.604 |
| [60,70[ | -0.15 | 1.25 | 0.904 |
| >=70 | -0.22 | 1.24 | 0.859 |
| **DISTH** (km) | -0.02 | 0.006 | <0.001 |
| **EDU** (years) | 0.22 | 0.05 | <0.001 |
| ***Low-AHA** |  |  |  |
| Medium-AHA | 0.65 | 0.37 | 0.075 |
| High-AHA | 2.16 | 0.36 | <0.001 |
| ***MST: Married** |  |  |  |
| Living together | 3.12 | 0.6 | <0.001 |
| Single | 0.25 | 0.49 | <0.001 |
| Widow | -0.14 | 0.34 | 0.678 |
| Divorced | 3 | 0.77 | <0.001 |
| ***VA: 0** |  |  |  |
| 0.1 | -0.74 | 0.6 | 0.218 |
| 0.2 | -2.14 | 0.58 | <0.001 |
| 0.3 | -1.99 | 0.63 | 0.002 |
| 0.4 | -0.94 | 0.54 | 0.084 |
| 0.5 | -0.29 | 0.53 | 0.58 |
| **CCI** | 0.18 | 0.52 | 0.729 |
| ***Aetiologies: AMD** |  |  |  |
| Diabetic retinopathy | 0.88 | 0.41 | 0.031 |
| Glaucoma | 0.6 | 0.55 | 0.271 |
| other | 0.33 | 0.44 | 0.5 |

***Reference category**
