# Supplementary material for: Predicting participation of people with impaired vision in epidemiological studies
Source: BMC Ophthalmol. 2018 Sep 4;18:236. doi: 10.1186/s12886-018-0889-9 (PMC6123934; doi:10.1186/s12886-018-0889-9)
Supplement: Supplementary file 2 — Table S2. The table summarizes new categories that were defined after having run the first logistic regression. The categories were used for our final model. (DOCX 17 kb) [file 12886_2018_889_MOESM2_ESM.docx]

**Table S2: The table summarizes new categories that were defined after having run the first logistic regression. The categories were used for our final model.**

| **Variables** | **Categories (levels)** |
| --- | --- |
| **Gender** | - Male  - Female |
| **DISTH** (km) | - Continuous |
| **EDU** (years) | - Continuous |
| **AHATTEND** | - AHA-rare = number of hospital visits was less than 10 per year;  - AHA-frequent = number of hospital visits was 10 or more per year; |
| **MST** | - Living together  - Divorced  - Others (married, single or widowed) |
| **VA** | - VA-extreme (includes VA of 0.0 or 0.1 or 0.5)  - VA-intermediate (includes VA of 0.2 or 0.3 or 0.4) |
